# Electroacupuncture preconditioning and mitochondria-associated outcomes in animal models of myocardial ischemia-reperfusion injury: a systematic review and meta-analysis

**DOI:** 10.3389/fcvm.2026.1982708

**Published:** 2026-10-30

**Authors:** Mingyi Du, Weiping Chen, Jianan Chen, Anran Liu, Jialin Jiang, Taochun Ye

**Affiliations:** 1 The First Clinical Medical School of Guangzhou University of Chinese Medicine, Guangzhou, China; 2 Changhua Subdistrict Community Health Service Center, Guangzhou, China; 3 The First Affiliated Hospital of Guangzhou University of Chinese Medicine, Guangzhou, China; 4 Guangdong Clinical Research Academy of Chinese Medicine, Guangzhou, China; 5 Shenzhen Longhua Hospital of The First Affiliated Hospital of Guangzhou University of Chinese Medicine, Shenzhou, China

**Keywords:** acupuncture preconditioning, meta-analysis, mitochondria-associated outcomes, myocardial ischemia-reperfusion injury, preclinical studies

## Abstract

**Background:**

Mitochondrial dysfunction is central to myocardial ischemia-reperfusion injury (MIRI). Although acupuncture preconditioning has shown cardioprotection in animal models, no systematic review has quantitatively synthesized mitochondria-associated outcomes.

**Objective:**

To evaluate the effects of acupuncture preconditioning on prespecified mitochondria-associated outcomes—reactive oxygen species (ROS), malondialdehyde (MDA), adenosine triphosphate (ATP), Bax, B-cell lymphoma 2 (Bcl-2), and apoptotic index (AI)—and infarct size in animal MIRI models, and to explore the potential mechanisms underlying its cardioprotective effects.

**Methods:**

Eight databases were searched from inception to 26 July 2026. Randomized controlled animal studies of acupuncture preconditioning in MIRI were included. Two reviewers screened studies, assessed risk of bias (SYRCLE), and extracted data; graphical data were digitized by one reviewer and verified by another. Random-effects meta-analyses used standardized mean differences (SMDs) with Hartung-Knapp-Sidik-Jonkman adjustment.

**Results:**

Thirty-four publications involving 586 animals were included: 33 electroacupuncture and 1 manual acupuncture. Thirty-three studies were pooled, and one study was qualitatively synthesized because its outcome direction could not be harmonized. Acupuncture preconditioning (predominantly electroacupuncture) reduced infarct size (SMD=-2.69, 95% CI −3.57 to −1.82, I^2^ = 72.9%), MDA (−3.76, −6.22 to −1.29, I^2^ = 86.8%), ROS (−4.02, −7.18 to −0.85, I^2^ = 92.1%), Bax (−6.52, −11.43 to −1.62, I^2^ = 93.7%), and AI (−6.36, −10.36 to −2.36, I^2^ = 96.5%), and increased ATP (3.21, 2.12 to 4.29, I^2^ = 69.9%) and Bcl-2 (8.14, 3.82 to 12.45, I^2^ = 94.7%). Subgroup analyses found significant effect modification only for Bcl-2 (species and reperfusion duration); heterogeneity remained high. Egger's test did not indicate publication bias, but trim-and-fill rendered ROS non-significant. Certainty was moderate for most outcomes and low for Bax.

**Conclusion:**

Electroacupuncture preconditioning was associated with reduced myocardial injury and favorable mitochondria-associated changes in animal MIRI models; only one manual acupuncture study was included, so these findings should not be generalized to acupuncture as a whole. These findings cannot establish mitochondrial protection as a causal mechanism; other pathways may also contribute and were not synthesized. Extreme effect sizes for ROS, Bax, Bcl-2, and AI may be overestimated. Evidence is preclinical and hypothesis-generating; sham-controlled studies are needed.

**Systematic Review Registration:**

https://www.crd.york.ac.uk/prospero/display_record.php?ID=CRD420261477352, PROSPERO CRD420261477352.

## 1 Introduction

Myocardial ischemia-reperfusion injury (MIRI) refers to the paradoxical exacerbation of myocardial damage that occurs upon restoration of blood flow to ischemic myocardium. In clinical management of acute myocardial infarction, although percutaneous coronary intervention and thrombolytic therapy can promptly reestablish coronary perfusion, reperfusion itself may trigger cardiomyocyte death, microvascular dysfunction, and arrhythmias—complications that partially offset the benefits of revascularization (1, 2). In this context, identifying adjunctive therapeutic strategies that safely and multi-targetedly intervene in the pathogenic processes of MIRI holds substantial clinical relevance.

The pathophysiology of MIRI involves intricate crosstalk among oxidative stress, calcium overload, inflammatory responses, mitochondrial dysfunction, and activation of the intrinsic apoptotic pathway, with mitochondrial impairment being one of the key mechanisms (3, 4). During ischemia, mitochondrial oxidative phosphorylation is suppressed, leading to reduced adenosine triphosphate (ATP) synthesis. Upon reperfusion, a massive burst of reactive oxygen species (ROS) ensues, triggering lipid peroxidation and elevating malondialdehyde (MDA) levels. Concurrently, opening of the mitochondrial permeability transition pore results in loss of membrane potential and release of pro-apoptotic factors, which in turn activate the Bax/Bcl-2-mediated apoptotic cascade and ultimately elevate the apoptotic index (AI) (5, 6).

Acupuncture preconditioning—defined as the application of specific acupoint stimulation prior to ischemia—has been shown to induce endogenous protective effects that mitigate subsequent myocardial injury caused by ischemia-reperfusion. Animal studies have demonstrated that acupuncture preconditioning reduces myocardial infarct size and exerts protective modulation on the aforementioned mitochondrial-related parameters, suggesting that mitochondrial protection may constitute one of its pivotal mechanisms (7–9).

Although several meta-analyses have examined the effects of acupuncture on MIRI, all harbor notable limitations. The investigations by Xiong et al. and Qin et al. were both based on clinical studies, thereby precluding elucidation of tissue- and molecular-level mechanisms (10, 11). Yang et al. conducted an animal study, but restricted their analysis to a single acupoint of electroacupuncture, failed to systematically integrate mitochondrial-related outcomes, and limited their literature search to publications up to 2014 (12). To date, no systematic review has comprehensively synthesized the evidence on acupuncture preconditioning in animal MIRI models specifically from a mitochondria-associated outcome perspective.

To address this gap, the present study systematically retrieved randomized controlled trials of acupuncture preconditioning in animal models of MIRI, and performed a meta-analysis to evaluate its effects on myocardial infarct size and mitochondria-associated parameters (MDA, ATP, ROS, Bax, Bcl-2, and AI). The overarching aim was to summarize the effects of acupuncture preconditioning on prespecified mitochondria-associated outcomes and infarct size in animal MIRI models, and to explore mitochondria-associated mechanisms as potential explanations.

## 2 Materials and methods

This review was registered with PROSPERO (CRD420261477352) on 13 August 2026. The last systematic search was completed on 26 July 2026, before registration; the registration is therefore described as retrospective with respect to the search stage. No primary outcomes or primary analysis plan were changed after registration. Deviations were more restrictive eligibility criteria (at least one mitochondria-associated outcome required; infarct size remained a secondary outcome) and non-duplicate digitization of graphical data. Both are summarized in Supplementary Table S1. The conduct and reporting of this systematic review and meta-analysis followed the most recent PRISMA (Preferred Reporting Items for Systematic Reviews and Meta-Analyses) guidelines to ensure methodological rigor (13) (Supplementary Table S2).

### 2.1 Data sources and search strategy

Systematic searches were conducted in the following electronic databases from inception to 26 July 2026: PubMed, Embase (via Ovid), the Cochrane Library, Web of Science, China National Knowledge Infrastructure (CNKI), VIP Chinese Science and Technology Periodical Database, Wanfang Database, and Chinese Biomedical Literature Database (CBM). No language or publication date restrictions were imposed.

Search terms were built around two conceptual blocks—"acupuncture” and “myocardial ischemia-reperfusion injury"—using a combination of Medical Subject Headings (MeSH) and free-text words. An illustrative search string was: {[Acupuncture Therapy(Mesh)] OR [Acupuncture Treatment(Title/Abstract)]} AND {[Myocardial Reperfusion Injury(Mesh)] OR [Injuries, Myocardial Reperfusion(Title/Abstract)]}. The detailed search strategy for each database is provided in Additional file 1.

### 2.2 Inclusion criteria

Studies were selected according to the PICOS framework, with the following criteria applied: ① Participants: Rats or mice subjected to MIRI, irrespective of the specific modeling method employed; ② Intervention: Acupuncture preconditioning with manual acupuncture or electroacupuncture alone, administered prior to ischemia, without restriction on acupoint selection or stimulation parameters; ③ Comparator: A concurrent untreated model control or a sham/non-acupoint/minimal acupuncture control, with no other active intervention in either group; ④ Outcomes: At least one of the following myocardial tissue parameters was required to be reported—ROS, MDA, ATP, Bax protein, Bcl-2 protein, or the AI; ⑤ Study design: Randomized controlled animal experiments; ⑥ Publication type: No language restriction was applied during the search; however, only original research articles published in English or Chinese were eligible. Reviews, conference abstracts, dissertations or theses, and duplicate publications were excluded.

### 2.3 Exclusion criteria

Studies were excluded if they met any of the following criteria: (1) non-randomized controlled studies; (2) animal experiments performed on species other than rats or mice; (3) interventions other than acupuncture; (4) protocols not involving preconditioning; (5) absence of a concurrent control meeting the inclusion criteria; (6) failure to report any of the aforementioned myocardial tissue outcomes; (7) incomplete or unextractable data; (8) non-original research publications, including reviews, conference abstracts, and dissertations or theses.

### 2.4 Data extraction

Two investigators (M.Y.D. and W.P.C.) independently extracted tabular and text data from the included studies using a standardized Excel spreadsheet and subsequently cross-checked their extractions. Graphical data were digitized by the first investigator (M.Y.D.) and verified by the second investigator (W.P.C.) against the original figures. Disagreements were resolved through discussion and consensus; if consensus could not be reached, a third investigator (T.C.Y.) made the final decision. This protocol deviation from the PROSPERO registration, which specified independent duplicate data extraction, is acknowledged as a limitation.

The following information was extracted from each eligible study: (1) basic study characteristics, including the first author's name and year of publication; (2) animal model features, encompassing species, strain, sex, body weight, modeling method, ischemic duration, and reperfusion duration; (3) intervention details, including acupuncture type, acupoint names, electroacupuncture parameters, duration of each stimulation session, and total number of preconditioning sessions, all of which were required to be administered prior to ischemia; (4) outcome data, including sample size per group, as well as mean values and standard deviations for each outcome measure. The outcomes of interest were restricted to myocardial tissue parameters, specifically infarct size, ROS, MDA, ATP, Bax and Bcl-2 protein expression levels, and the apoptotic index.

When SEM was reported, SD was calculated as SD = SEM × √n. All continuous outcomes were analyzed using SD.

For studies reporting data only in figures, values were digitized once by the first investigator(M.Y.D) using GetData Graph Digitizer. The x- and y-axes were calibrated according to the original axis labels, and the figure was magnified before extraction. Digitized values were verified by the second investigator(W.P.C) against the original figure, figure legend, and available text or tables. If a discrepancy or suspected error was identified, the figure was recalibrated and re-extracted; unresolved discrepancies were resolved by consensus or by a third investigator(T.C.Y). Because independent duplicate digitization was not performed, digital measurement error cannot be completely excluded. For studies with incomplete data reporting, the corresponding authors were contacted via email to request supplementary information. If the required data remained unavailable after contact, the study was excluded during the screening phase, with the reason for exclusion documented in the PRISMA flow diagram.

### 2.5 Risk of bias assessment of included studies

Two investigators (M.Y.D. and W.P.C.) independently assessed the methodological quality of the included studies using the Systematic Review Centre for Laboratory Animal Experimentation (SYRCLE) Risk of Bias tool for animal studies. This tool, adapted from the Cochrane Risk of Bias instrument, comprises 10 items covering six domains: selection bias, performance bias, detection bias, attrition bias, reporting bias, and other bias (14). Each item was determined to have a low, high, or unclear risk of bias based on the corresponding signaling questions. The specific items were: (1) selection bias (sequence generation, baseline comparability, and allocation concealment); (2) performance bias (random housing and blinding of investigators); (3) detection bias (random outcome assessment and blinding of assessors); (4) attrition bias (incomplete outcome data); (5) reporting bias (selective outcome reporting); and (6) other bias. The assessment results were presented in graphical formats, including a risk-of-bias summary plot and a bar chart displaying the proportion of studies for each risk category.

### 2.6 Quality of evidence assessment

The certainty of evidence for each outcome was graded using the Grading of Recommendations Assessment, Development and Evaluation (GRADE) system (15, 16). Two investigators independently performed the rating, with disagreements resolved by a third investigator. Downgrading considerations included risk of bias, inconsistency, indirectness, imprecision, and publication bias. Risk of bias was determined based on the SYRCLE assessment results. Inconsistency was evaluated by examining the consistency of effect directions across studies and the degree of overlap of confidence intervals; no automatic downgrading was applied when effect directions were consistent, and I^2^ was considered only as a supplementary reference. This review addressed a preclinical question in animal models; therefore, animal studies were considered direct evidence and indirectness was not downgraded. However, these findings remain indirect for clinical patients and should not be directly extrapolated. Imprecision was judged based on the width of confidence intervals and the total sample size. Publication bias was assessed using funnel plots and Egger's test when ten or more studies were available for an outcome; when fewer than ten studies were available, publication bias was not assessed. The GRADE results were presented in a summary of findings table.

### 2.7 Statistical analysis

Meta-analyses were conducted using R software (version 4.5.1), with data pooling and statistical computations performed via the metafor package. All outcome measures were continuous variables, and the standardized mean difference (SMD) with its 95% confidence interval (CI) was uniformly used as the effect size. Heterogeneity was assessed using the I^2^ statistic, with I^2^ ≥ 50% considered indicative of substantial heterogeneity. Given the anticipated heterogeneity inherent in animal experiments—arising from variations in species, modeling methods, and intervention protocols—a random-effects model was adopted by default, and confidence intervals were estimated using the Hartung-Knapp-Sidik-Jonkman (HKSJ) method. Prespecified subgroup factors were animal species, modeling method, acupuncture type (EA vs. MA), treatment course (number of preconditioning sessions), ischemia duration, reperfusion duration, and animal sex; each subgroup analysis required at least two studies. Subgroup analyses were performed only when this condition was met, and factors that could not be analyzed are reported in the Results. Sensitivity analysis was conducted by sequentially excluding individual studies to evaluate the robustness of the pooled effect estimates. When ten or more studies were available for a given outcome, publication bias was assessed using funnel plots in conjunction with Egger‘s test. Because SMD was used as the effect measure, Egger's test was performed with the inverse of the square root sample size (1/√n) as the predictor to avoid the inherent mathematical correlation between effect size and standard error. Trim-and-fill analysis used a random-effects model. As a *post hoc* analysis, prediction intervals were calculated using the non-parametric method proposed by Wang and Lee (17), because parametric prediction intervals require a large-sample approximation (18), similar study sizes (19), and normally distributed random effects. The non-parametric method does not rely on these assumptions and is recommended when sample sizes are small or normality is unrealistic (20). Additional exploratory analyses examined the influence of publication language and research team clustering.

## 3 Results

### 3.1 Study selection

Two investigators (W.P.C. and M.Y.D.) searched eight electronic databases and identified 1,188 records. After 741 duplicates were removed, 447 records were screened by title and abstract; 298 were excluded, leaving 149 full-text articles for eligibility assessment. Of these, 115 were excluded, and 34 publications were included in the qualitative synthesis. Thirty-three publications were included in the meta-analysis. One study (21) was retained in the qualitative synthesis only: although it met the eligibility criteria, its outcome was measured by a different assay in which the direction of beneficial change was opposite to that in the other studies, and the data could not be harmonized into a common direction for pooling. All 33 studies included in the meta-analysis contributed data to at least one outcome. The screening process is shown in Figure 1. Among these 33 publications, two pairs of reports were suspected to be duplicate reports: Chen et al. (22) and Chen et al. (23), and Tan et al. (24) and Du et al. (25). These pairs shared overlapping authors, institutions, animal models, acupuncture protocols, and outcome data. Because duplication could not be definitively confirmed, both reports in each pair were retained as separate reports in the primary analysis, and sensitivity analyses excluding both reports in each suspected pair were performed. (Chen et al. pair: ATP; Tan/Du pair: AI).

**Figure 1:**
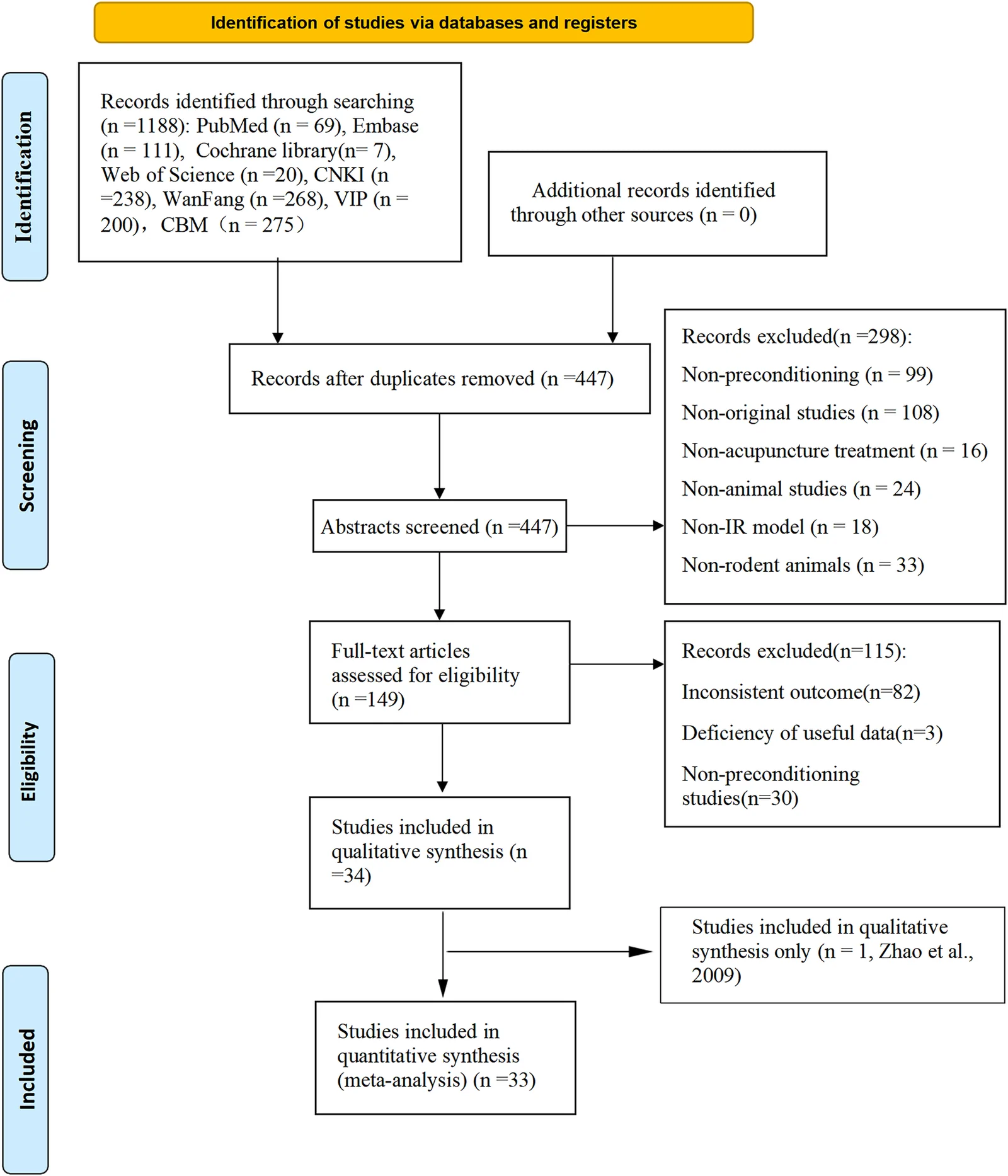
PRISMA 2020 flow diagram of the study selection process.

### 3.2 Characteristics of included studies

A total of 34 publications were ultimately included in this review (8, 21–53), comprising 14 English-language and 20 Chinese-language studies, all of which were randomized controlled animal experiments published between 2009 and 2026. Among the 33 studies included in the meta-analyses, 19 (57.6%) required digital extraction from figures. Acupuncture preconditioning served as the intervention in all included studies, with 33 employing electroacupuncture and one using manual acupuncture. Regarding acupoint selection, the most frequently used regimen was PC6 alone, adopted in 20 studies (58.8%), followed by PC6 in combination with other acupoints in 10 studies (29.4%), and non-PC6 acupoints—including BL15, HT7, LI4, and Ningshen—in the remaining 4 studies (11.8%). The most common preconditioning protocol consisted of 20-minute sessions administered once daily for seven consecutive days. Among electroacupuncture parameters, continuous wave at 2 Hz was most frequently applied, with intensity typically set at 1 mA, whereas a sparse-dense wave was adopted in a subset of studies.

In terms of animal models, Sprague-Dawley (SD) rats were the predominant species, used in 15 studies (44.1%), followed by Wistar rats in 13 studies (38.2%), and C57BL/6 mice in 3 studies (8.8%). The remaining 3 studies employed other strains, including Stat5fl/fl mice, Kunming mice, and mice with unspecified strains. Myocardial ischemia-reperfusion injury was induced via left anterior descending coronary artery ligation in 33 studies (97.1%), whereas the remaining study utilized the Langendorff isolated heart perfusion model. Ischemic durations ranged from 20 to 40 min, and reperfusion durations from 30 to 4,320 min. All studies included a concurrent model control group that did not receive acupuncture treatment, with sample sizes ranging from 3 to 20 animals per group.

Among the outcomes of interest, the AI was the most frequently reported, documented in 14 studies, followed by infarct size in 13 studies, ROS in 10, Bcl-2 in 10, ATP in 8, Bax in 7, and MDA in 7. Detailed characteristics of the included studies are presented in Supplementary Table 3, and the distributions of animal species, outcomes, and acupoint regimens are illustrated in Figure 2.

**Figure 2:**
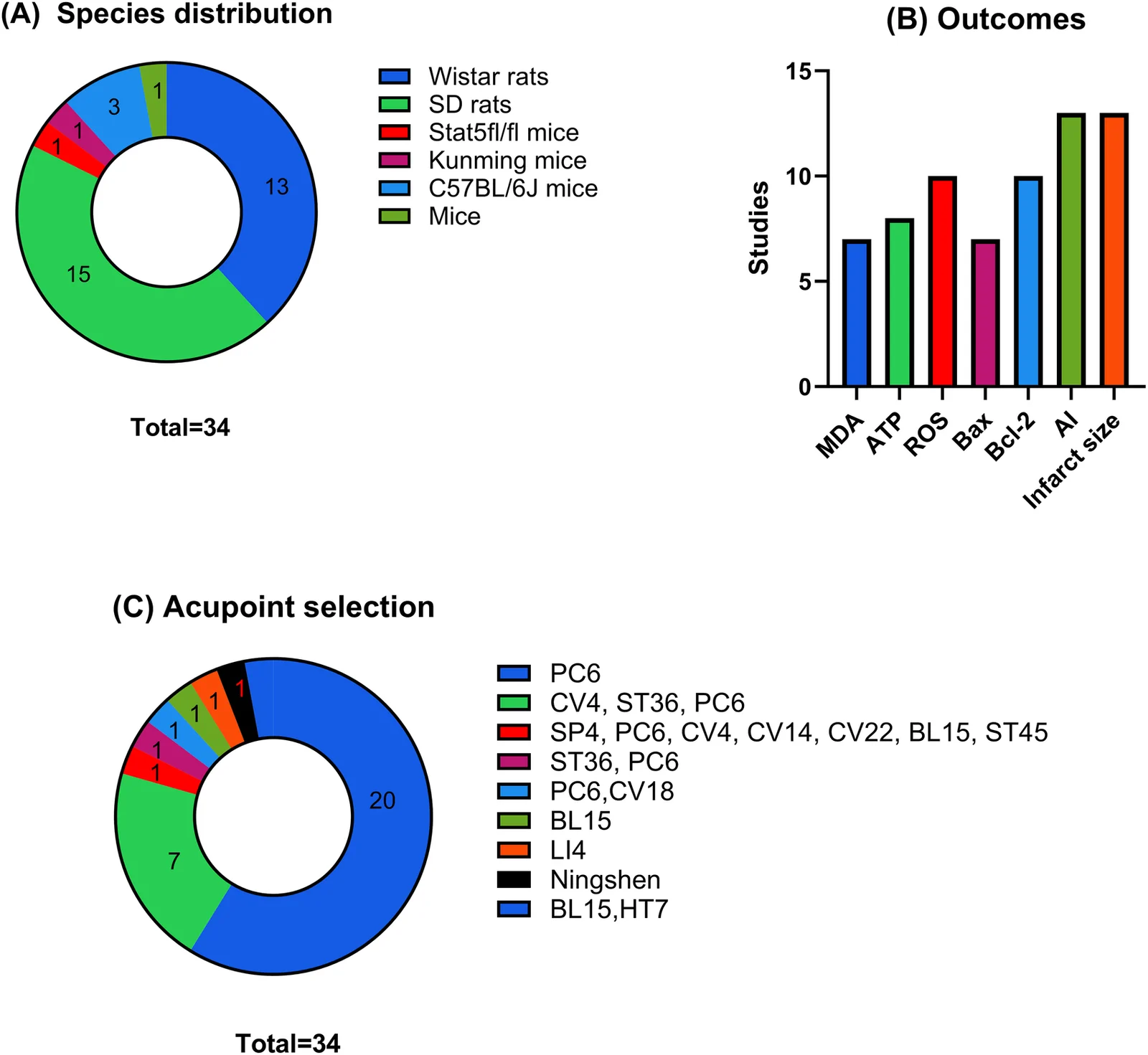
Characteristics of the included studies. **(A)** Species distribution **(B)** Outcomes **(C)** Acupoint selection.

### 3.3 Risk of bias

The methodological quality of the included studies was assessed at the domain level using the SYRCLE tool, without assigning a composite overall risk-of-bias category. Selective outcome reporting was rated as low risk in all studies. Most studies were rated as unclear for baseline characteristics, allocation concealment, random housing, and blinding of caregivers/researchers. Random outcome assessment was rated as low risk in 9 studies, and blinding of outcome assessors was rated as low risk in only 2 studies. Incomplete outcome data were rated as high risk in 1 study (23). The detailed domain-level judgments and the study-level summary are presented in Figures 3A,B, respectively.

**Figure 3:**
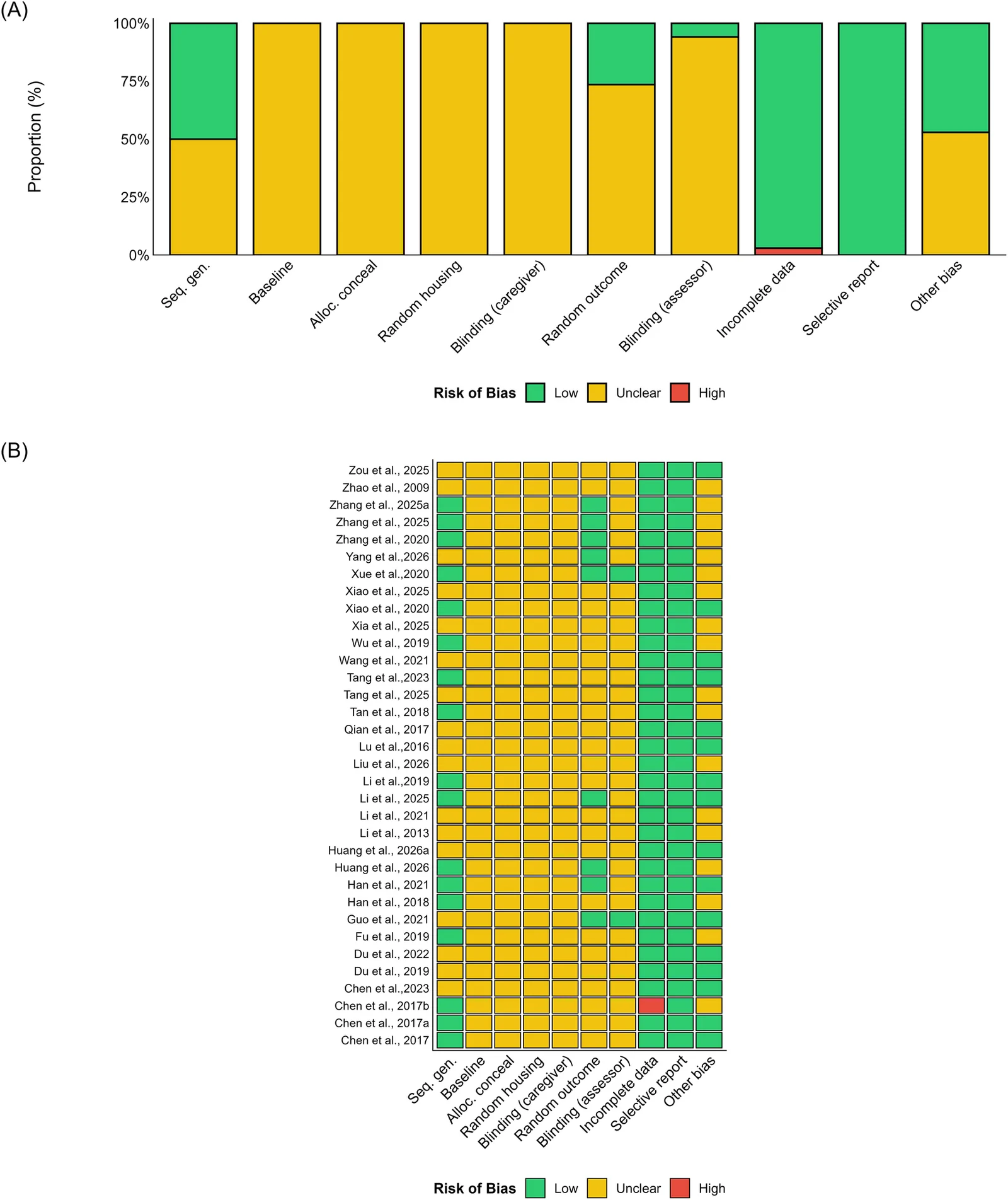
Risk of bias assessment using the SYRCLE tool. **(A)** Proportion of studies with low, unclear, and high risk of bias across domains. **(B)** Domain-level judgments for each included study.

### 3.4 Meta-analysis

Meta-analyses were conducted separately for the seven outcomes of interest across the 33 included studies. Collectively, acupuncture preconditioning exerted significant protective effects on mitochondrial-related parameters, as demonstrated by reduced MDA, ROS, Bax, and apoptotic index, elevated ATP and Bcl-2 levels, and reduced myocardial infarct size.

#### 3.4.1 Infarct size

Thirteen studies reported myocardial infarct size. Acupuncture preconditioning significantly reduced infarct size (SMD = −2.69, 95% CI: −3.57 to −1.82, 95% PI: −4.73 to −0.96, I^2^ = 72.9%, *P* *<* 0.0001), indicating that acupuncture preconditioning effectively attenuated the extent of irreversible myocardial tissue injury (Figure 4A).

**Figure 4:**
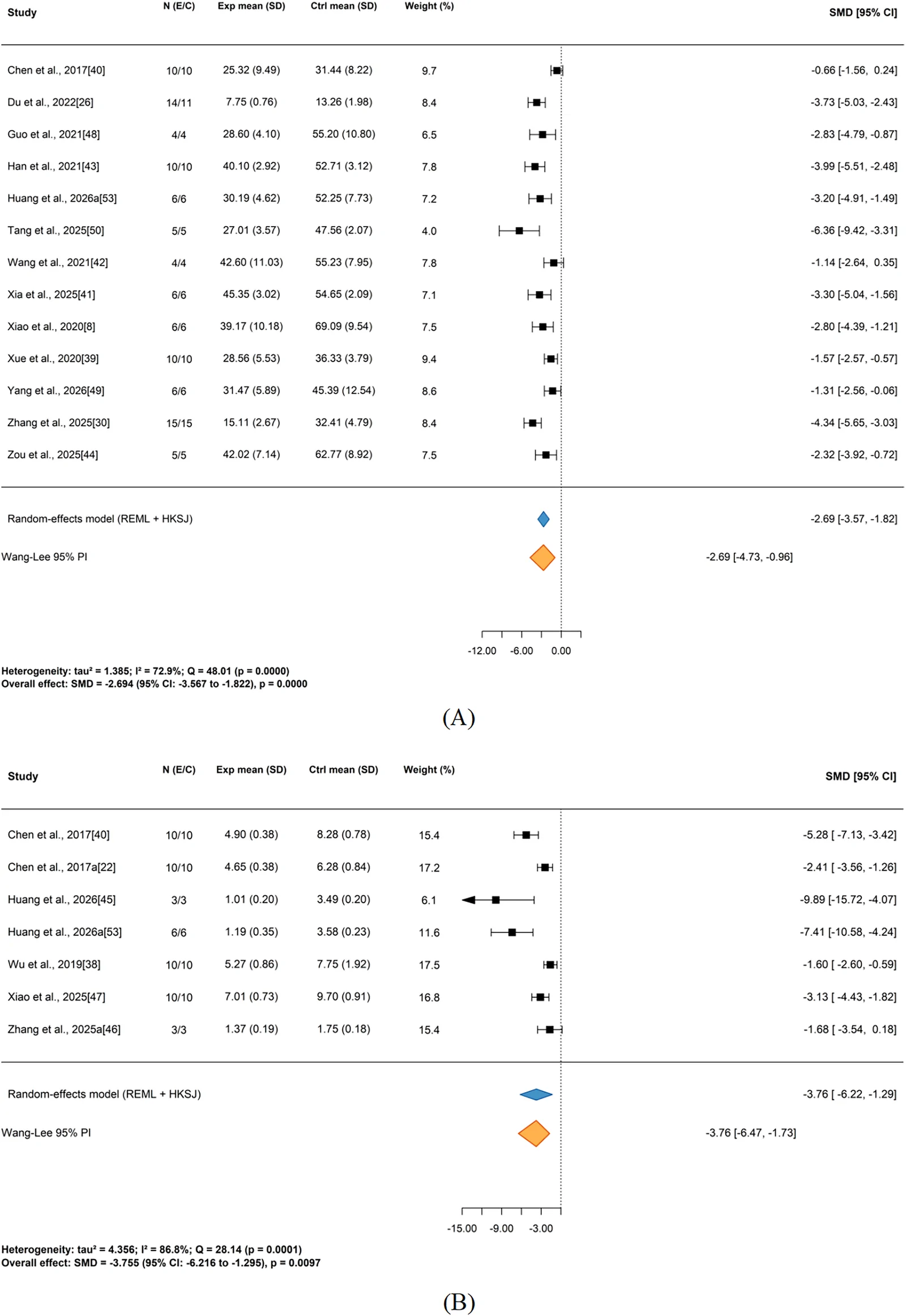
Forest plots for **(A)** infarct size and **(B)** MDA.

#### 3.4.2 MDA

Seven studies reported myocardial MDA levels. Acupuncture preconditioning significantly reduced MDA levels (SMD = −3.76, 95% CI: −6.22 to −1.29, 95% PI: −6.47 to −1.73, I^2^ = 86.8%, *P* *=* 0.0097), suggesting that acupuncture preconditioning attenuated lipid peroxidation injury (Figure 4B).

#### 3.4.3 ATP

Eight studies reported myocardial ATP content. Acupuncture preconditioning significantly elevated ATP levels (SMD = 3.21, 95% CI: 2.12 to 4.29, 95% PI: 1.81 to 4.56, I^2^ = 69.9%, *P* = 0.0002), demonstrating that acupuncture preconditioning improved myocardial energy metabolism following ischemia-reperfusion (Figure 5A).

**Figure 5:**
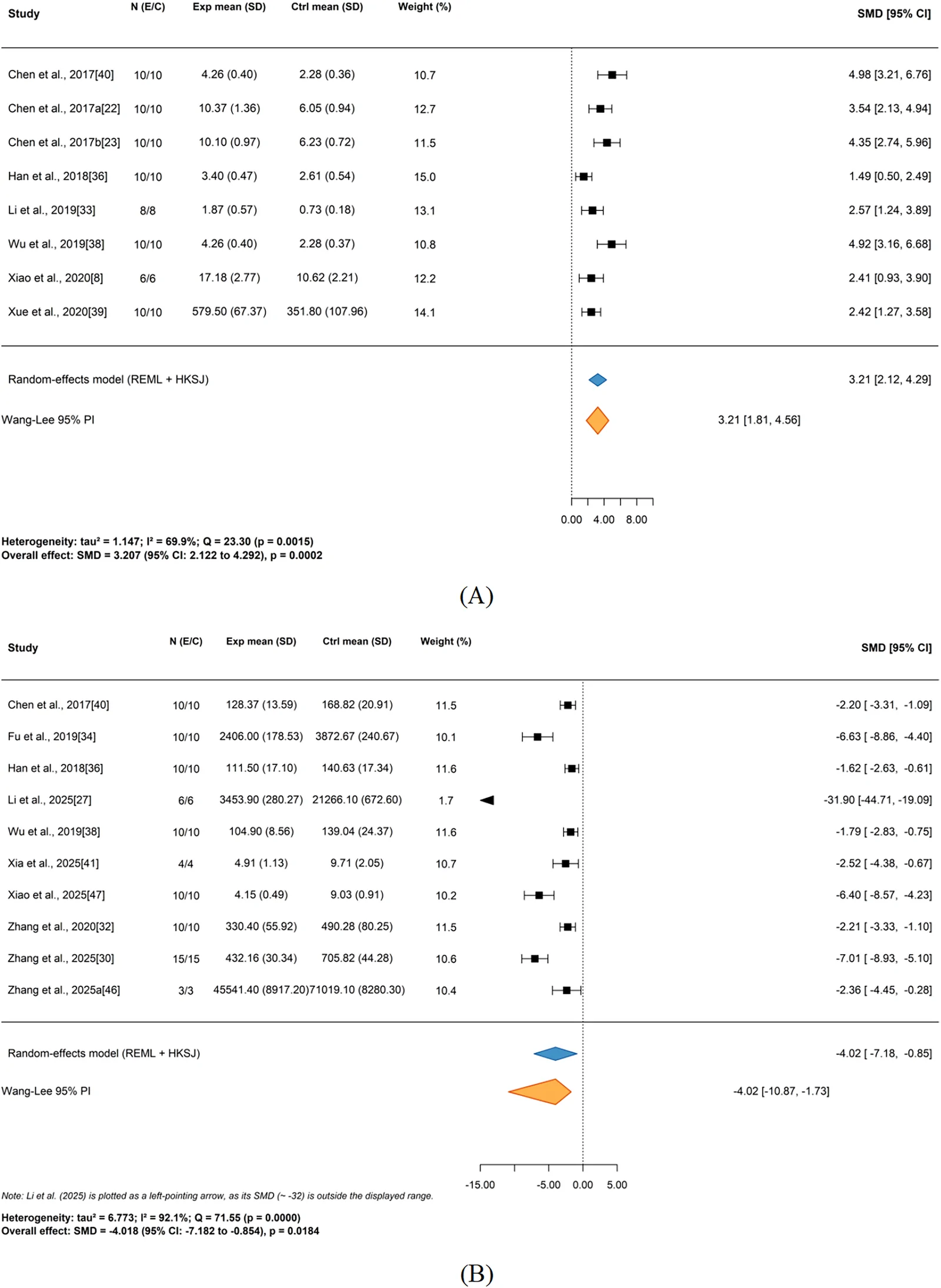
Forest plots for **(A)** ATP and **(B)** ROS.

#### 3.4.4 ROS

Ten studies reported myocardial ROS levels. Acupuncture preconditioning significantly reduced ROS levels (SMD = −4.02, 95% CI: −7.18 to −0.85, 95% PI: −10.87 to −1.73, I^2^ = 92.1%, *P* = 0.0184), suggesting that acupuncture preconditioning attenuated the oxidative stress burst during early reperfusion (Figure 5B).

#### 3.4.5 Bax

Seven studies reported myocardial Bax protein expression. Acupuncture preconditioning significantly downregulated Bax expression (SMD = −6.52, 95% CI: −11.43 to −1.62, 95% PI: −13.26 to −1.47, I^2^ = 93.7%, *P* = 0.01).74), indicating that acupuncture preconditioning inhibited the activation of pro-apoptotic signaling (Figure 6A).

**Figure 6:**
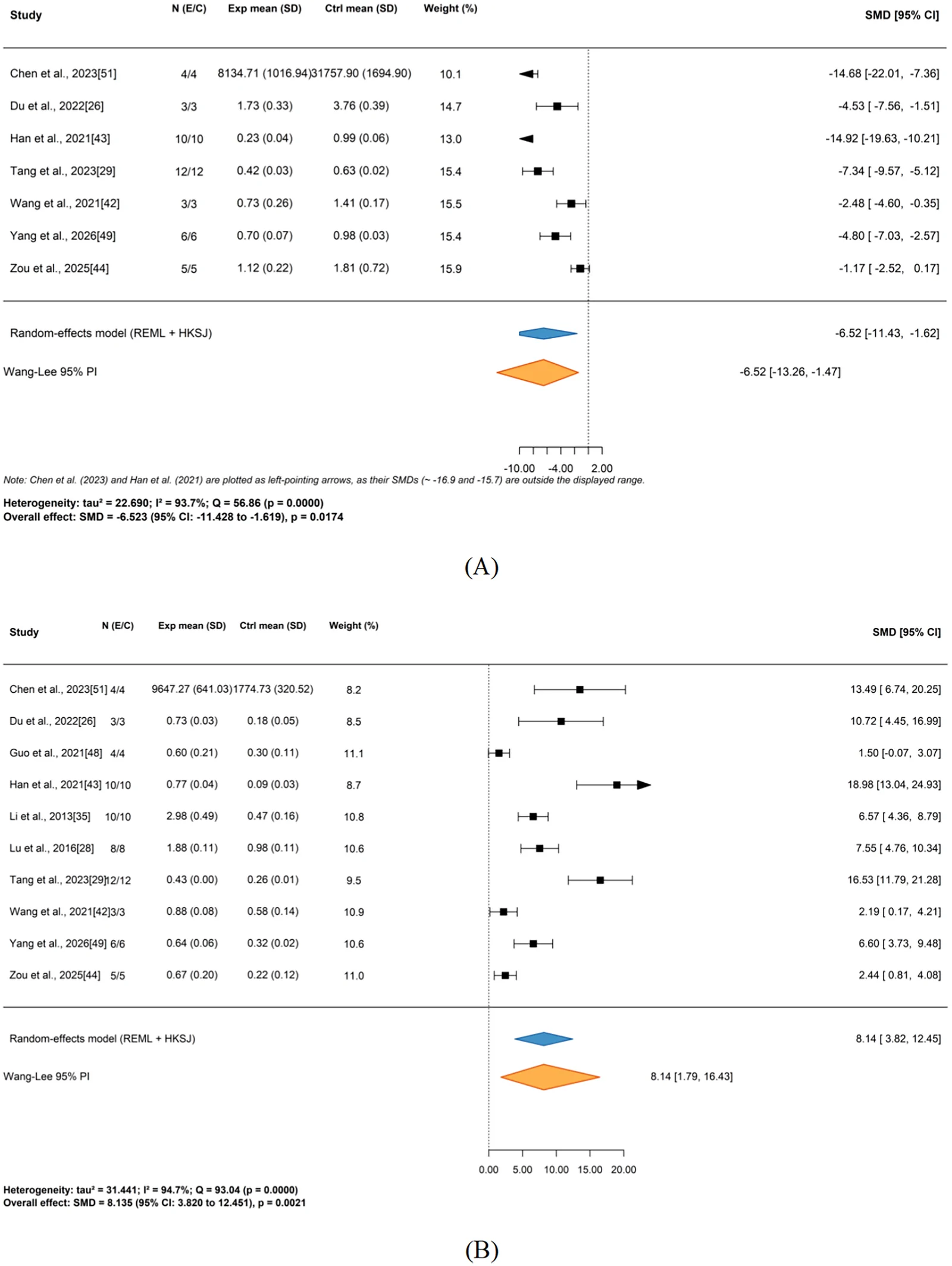
Forest plots for **(A)** Bax and **(B)** Bcl-2.

#### 3.4.6 Bcl-2

Ten studies reported myocardial Bcl-2 protein expression. Acupuncture preconditioning significantly upregulated Bcl-2 expression (SMD = 8.14, 95% CI: 3.82 to 12.45, 95% PI: 1.79 to 16.43, I^2^ = 94.7%, *P* = 0.0021), indicating that acupuncture preconditioning enhanced anti-apoptotic capacity, which together with Bax downregulation contributed to the suppression of the mitochondrial apoptotic pathway (Figure 6B).

#### 3.4.7 AI

Thirteen studies were included in the pooled analysis of the myocardial apoptotic index. Acupuncture preconditioning significantly reduced the apoptotic index (SMD = −6.36, 95% CI: −10.36 to −2.36, 95% PI: −16.29 to −1.69, I^2^ = 96.5%, *P* = 0.0047), suggesting that acupuncture preconditioning effectively inhibited cardiomyocyte apoptosis, an effect consistent with the suppression of the mitochondrial apoptotic pathway as reflected by Bax downregulation and Bcl-2 upregulation (Figure 7). One additional study (21) reported apoptosis as TUNEL average gray scale (higher = fewer apoptotic cells), the opposite direction to the pooled studies, and was therefore not combined; narratively, acupuncture increased this gray scale, indicating reduced apoptosis.

**Figure 7:**
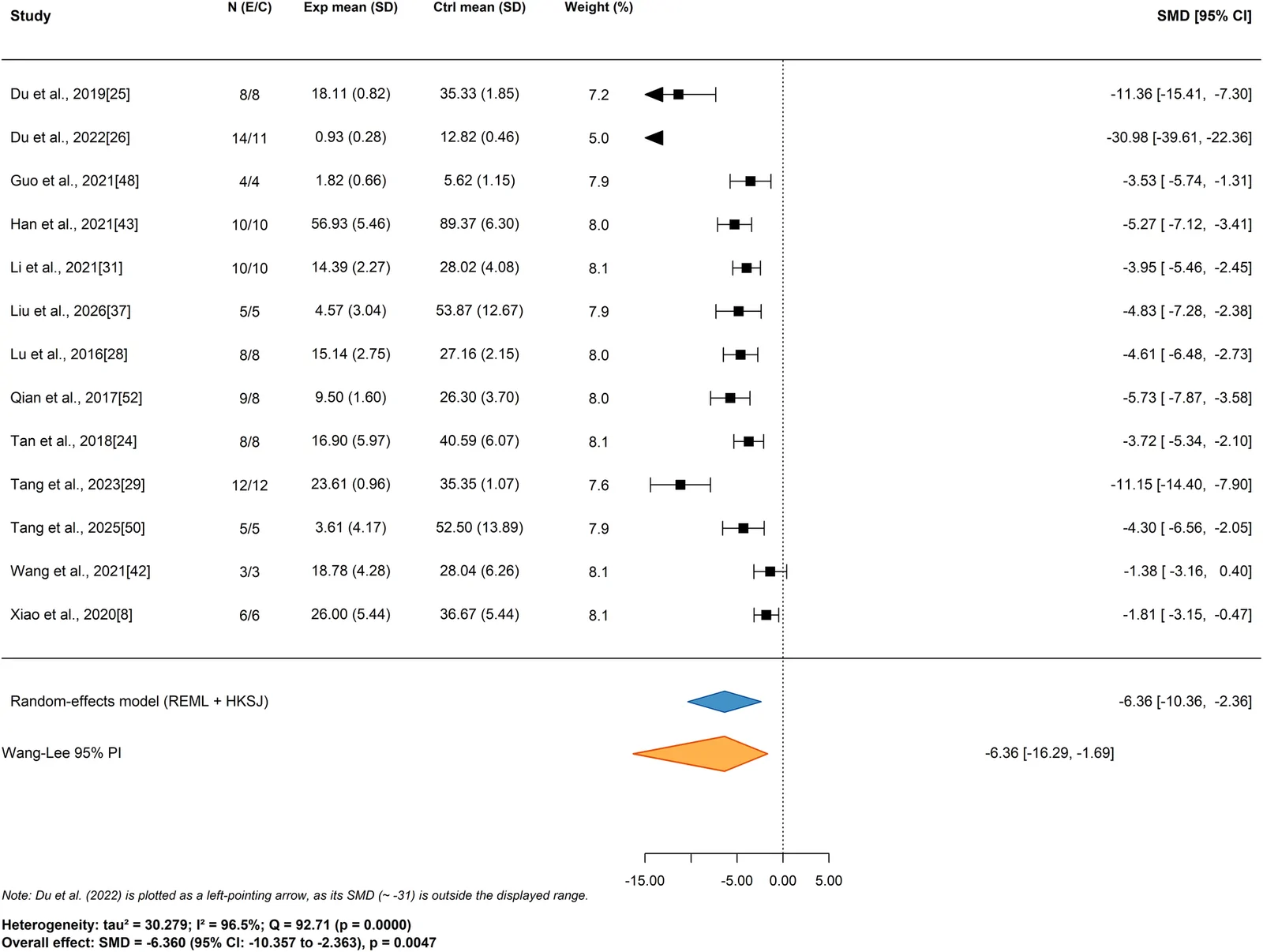
Forest plots for AI.

#### 3.4.8 Exploratory analysis using sham acupuncture controls

Three included studies also had a sham acupuncture control. For AI, two studies were pooled; acupuncture preconditioning reduced AI compared with sham acupuncture (SMD = −1.76, 95% CI −2.64 to −0.89, I^2^ = 0.0%, *P* = 0.0001). The remaining sham-controlled outcomes were each reported by only one study: ATP and infarct size favored acupuncture, ROS was reduced, and Bcl-2 was increased, but these findings could not be pooled. No sham-controlled data were available for MDA or Bax.

### 3.5 Subgroup analyses

Prespecified subgroup factors were animal species, modeling method, acupuncture type, treatment course, ischemia duration, reperfusion duration, and animal sex. Modeling method and acupuncture type were not analyzed because the alternative categories each contained only one study. Species subgroup analysis was feasible only for infarct size, Bax, Bcl-2, and AI. Between-subgroup differences were assessed using HKSJ-adjusted meta-regression. Detailed results are presented in Supplementary Table S4.

Subgroup analyses identified significant effect modification only for Bcl-2. Species significantly modified the effect (interaction *P* = 0.0124), with a larger effect in rats than in mice; however, within-subgroup I^2^ remained high in rats (84.8%) and was 0% in mice. Reperfusion duration also showed a significant between-subgroup difference for Bcl-2 (interaction *P* = 0.0238), with a larger effect in the short-reperfusion subgroup, but within-subgroup I^2^ remained high (82% and 88.1%). For all other outcomes, none of the analyzed subgroup factors significantly modified the effect sizes (all interaction *P* > 0.05), and heterogeneity remained substantial in most strata. Overall, the prespecified subgroup factors did not adequately explain the observed heterogeneity.

### 3.6 Publication bias

Publication bias was assessed using funnel plots (Figure 8) and Egger's test for outcomes with ten or more studies (infarct size, ROS, Bcl-2, and AI). The results are presented in Table 1. Egger's test did not suggest significant publication bias for any of these outcomes. For ROS, trim-and-fill analysis suggested one potentially missing study, and the adjusted pooled estimate became non-significant; this finding should be interpreted cautiously given the high heterogeneity. For outcomes with fewer than ten studies (MDA, ATP, and Bax), formal statistical assessment was not performed, and publication bias was rated as undetected rather than assumed to be absent.

**Figure 8:**
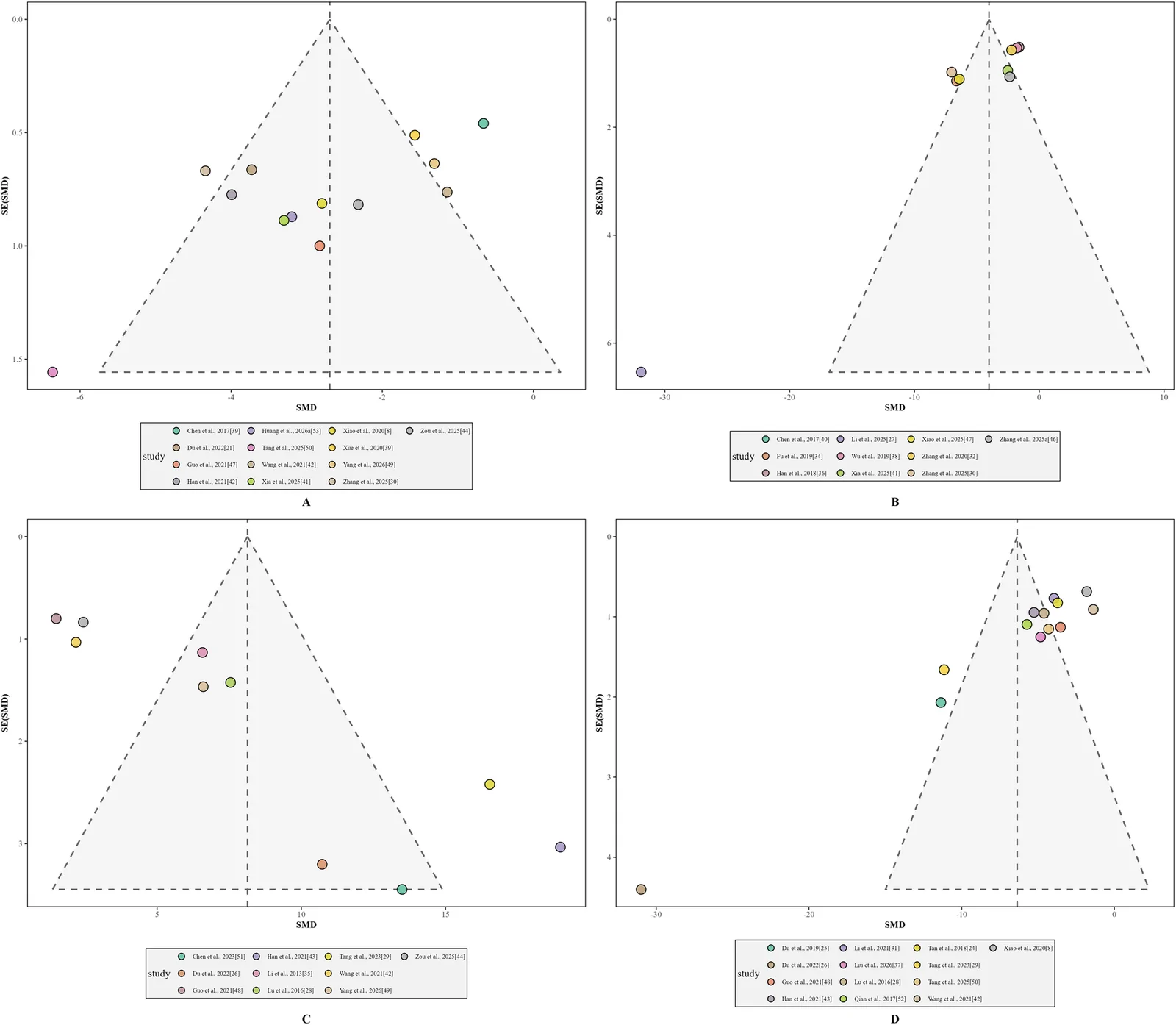
Funnel plots for publication bias assessment. **(A)** Infarct size, **(B)** ROS, **(C)** Bcl-2, and **(D)** AI.

**Table 1:** Egger's test and trim-and-fill method.

Outcomes	Egger's test	Before trim and fill			After trim and fill		
	*P*-value	*P*-value	EST	No. studies	*P*-value	EST	No. studies
Infarct size	0.5665	<0.0001	−2.69	13	<0.0001	−2.37	15
ROS	0.7597	0.0184	−4.02	10	0.1852	−3.61	11
Bcl-2	0.1408	0.0021	8.14	10	0.0021	8.14	10
AI	0.0818	0.0047	−6.36	13	0.0047	−6.36	13

Egger's test used the inverse of the square root sample size (1/√n) as the predictor; trim-and-fill used a random-effects model. Heterogeneity was high; for ROS, one missing study was estimated and the result became non-significant after trim-and-fill (*P* = 0.1852), which should be interpreted cautiously. Overall, no consistent evidence of publication bias was found. EST, Estimate.

### 3.7 Sensitivity analysis

Sensitivity analysis was conducted using the leave-one-out approach. The direction of the pooled effect sizes for all seven outcomes remained unchanged, with no 95% CIs crossing the line of no effect. However, for ROS, Bax, Bcl-2, and AI, omitting specific outlier studies led to noticeable reductions in the magnitude of the pooled SMDs. This indicates that while the overall statistical significance is robust, the extreme magnitudes of these effect sizes are highly sensitive to a few individual studies with extremely small standard deviations or methodological scale differences. The complete ranges of effect sizes and corresponding confidence intervals after sequential omission are detailed in Figure 9. In addition, two pairs of reports (22–25) were suspected to be duplicate reports. Because duplication could not be definitively confirmed, both reports in each pair were retained in the primary analysis, and *post hoc* sensitivity analyses were performed excluding both reports in each suspected pair simultaneously. The direction and statistical significance of the pooled effects for the relevant outcomes, ATP and AI remained unchanged after this exclusion, indicating that the primary findings were robust to the potential duplicate reports. Detailed results are presented in Figure 10. In exploratory sensitivity analyses, neither publication language nor research team clustering materially altered the overall conclusions. No significant language-by-treatment interaction was observed for any outcome (all P for interaction > 0.05; Figure 11), although some English subgroups were too small for stable estimates. In the leave-one-cluster-out analysis (Figure 12), infarct size, ATP, Bcl-2, and AI remained significant after excluding any single cluster, whereas MDA, Bax, and ROS were sensitive to specific clusters; excluding Hubei_UCM rendered MDA and ROS non-significant, and excluding Heilongjiang_UCM rendered Bax non-significant. These outcomes should therefore be interpreted with caution.

**Figure 9:**
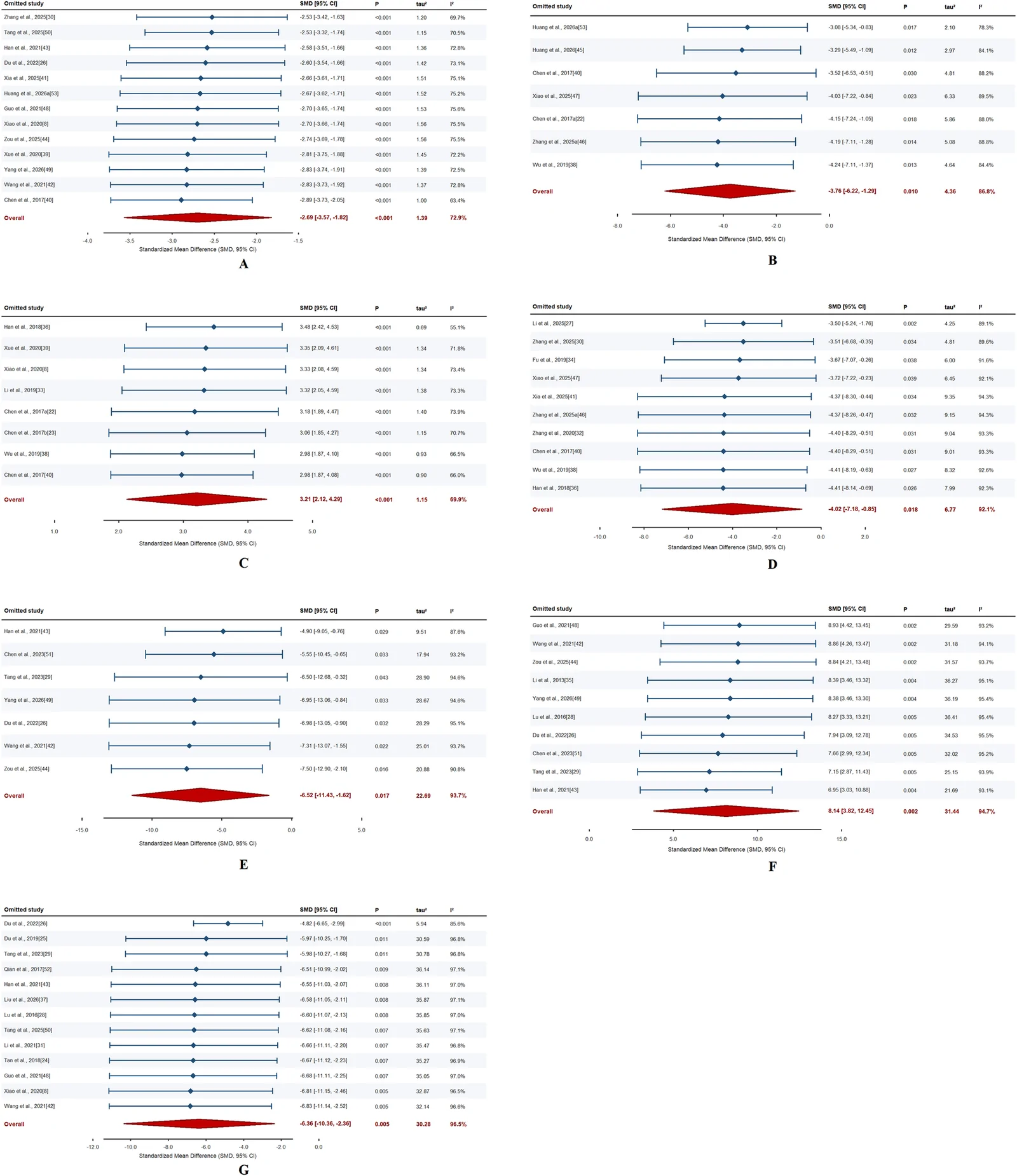
Leave-one-out sensitivity analysis across seven outcomes. **(A)** Infarct size; **(B)** MDA; **(C)** ATP; **(D)** ROS; **(E)** Bax; **(F)** Bcl-2; **(G)** AI.

**Figure 10:**
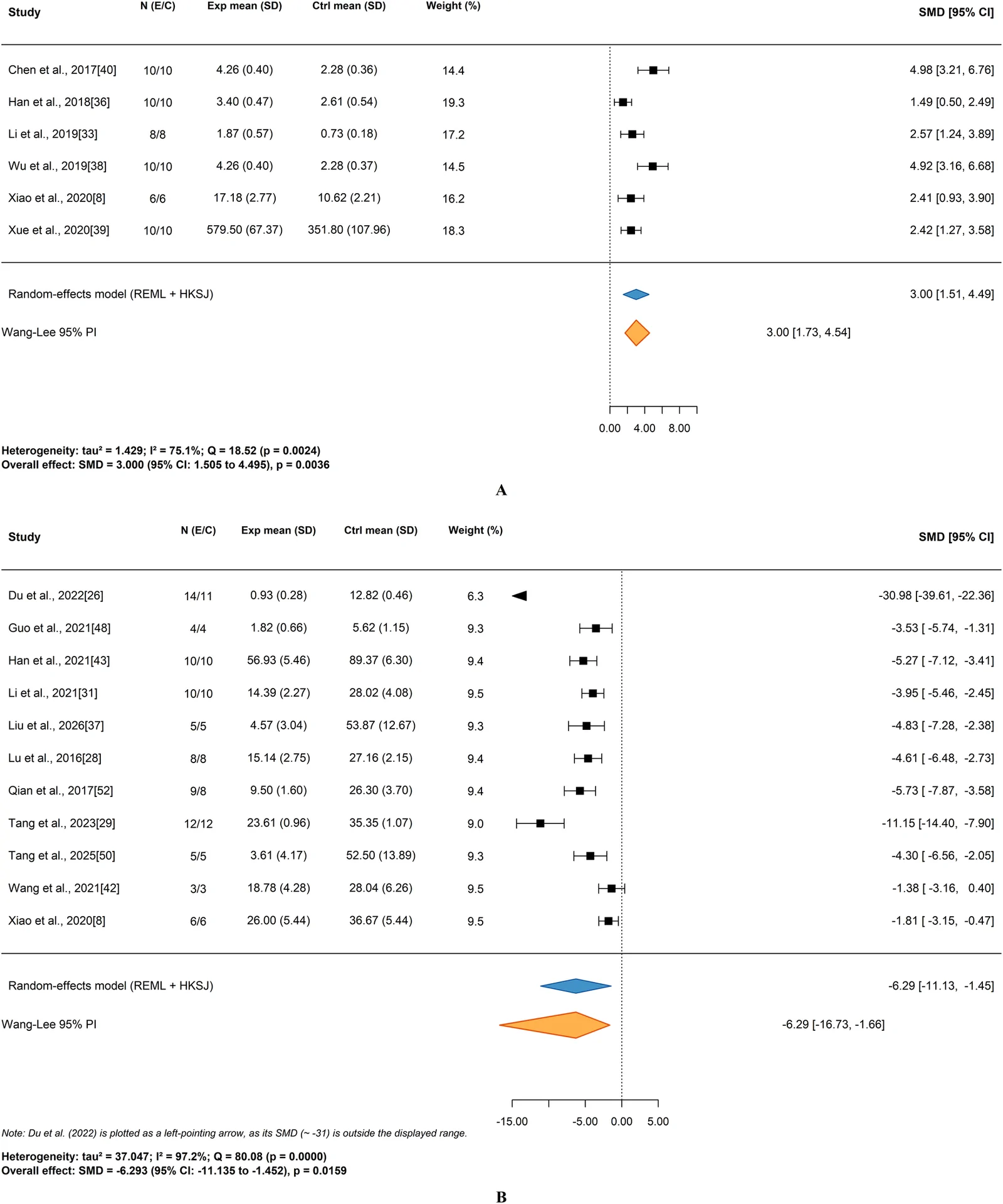
Sensitivity analysis excluding suspected duplicate reports: forest plots of acupuncture preconditioning on **(A)** ATP and **(B)** AI.

**Figure 11:**
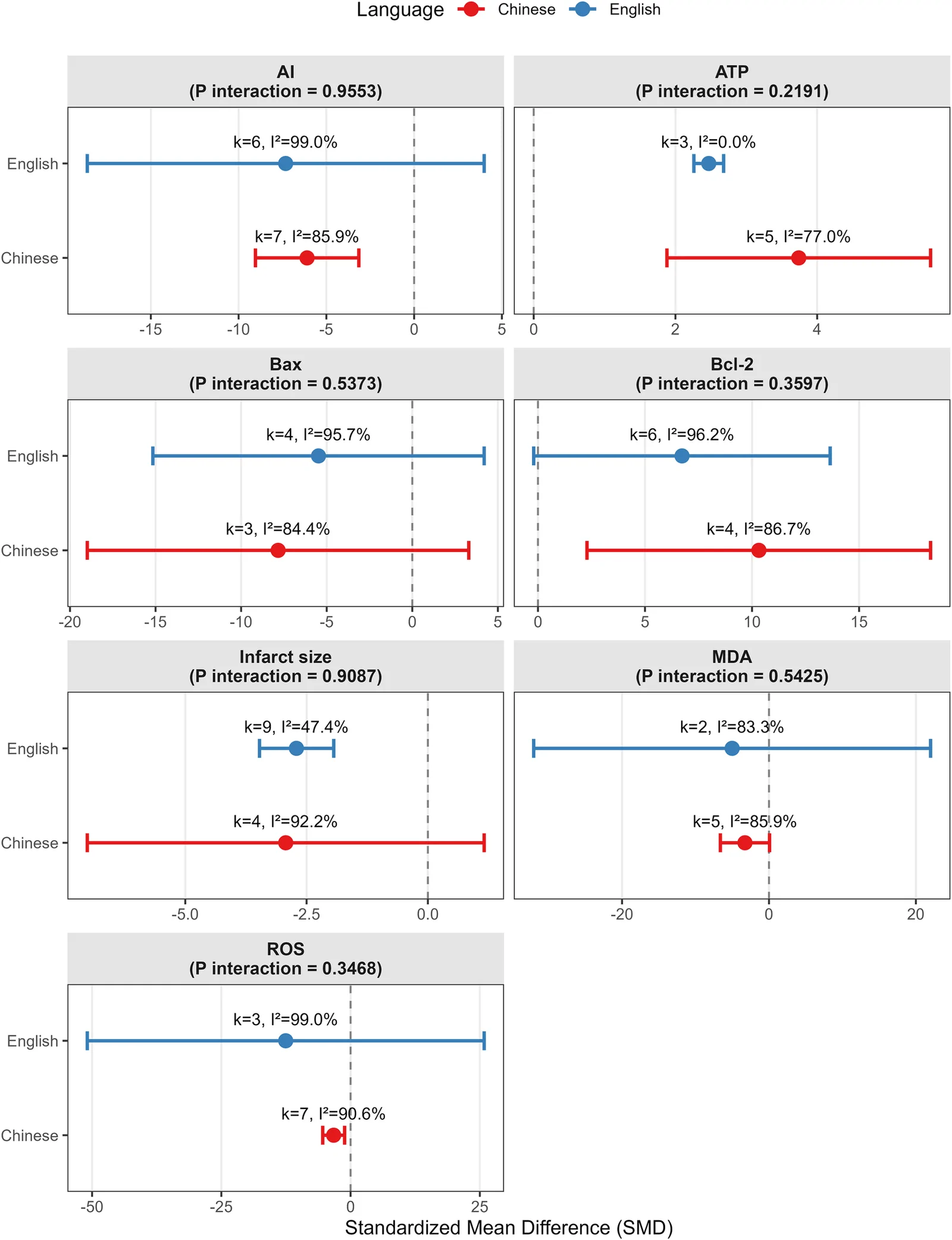
Sensitivity analysis by language of publication.

**Figure 12:**
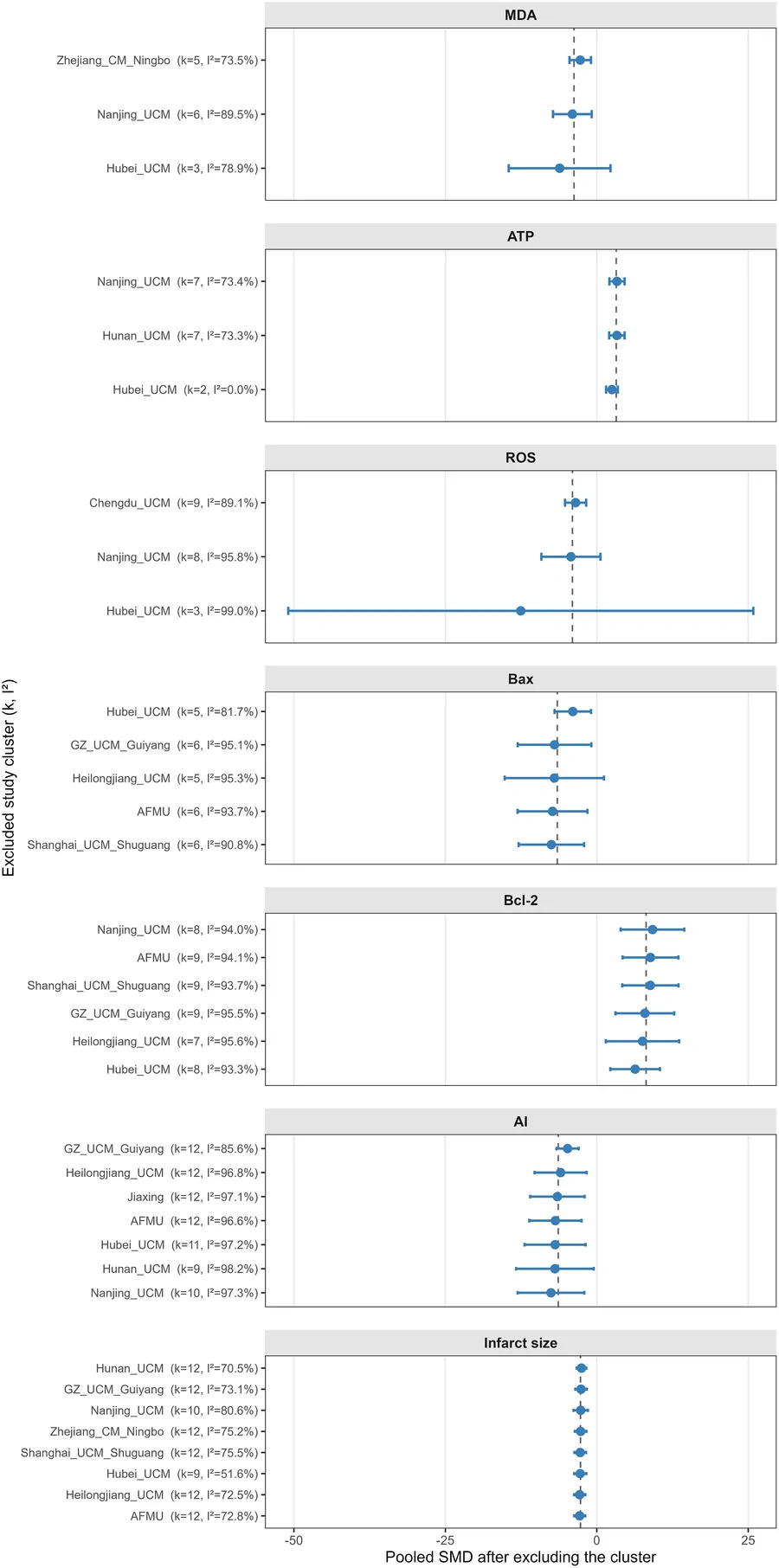
Sensitivity analysis by study cluster.

### 3.8 GRADE evidence assessment

The certainty of evidence for the seven outcomes was graded using the GRADE system. The certainty of evidence was moderate for MDA, ATP, ROS, Bcl-2, AI, and infarct size, and low for Bax. The detailed GRADE assessments and reasons for downgrading for each outcome are presented in Supplementary Table S5.

## 4 Discussion

This meta-analysis systematically evaluated the protective effects of acupuncture preconditioning in animal models of MIRI from multiple mitochondria-associated dimensions—energy metabolism (ATP), oxidative stress (ROS, MDA), and the mitochondrial apoptotic pathway (Bax, Bcl-2, AI). The results demonstrated that acupuncture preconditioning significantly reduced myocardial infarct size while concurrently improving the aforementioned mitochondrial-related parameters. Taken together, these findings suggest that acupuncture preconditioning may modulate mitochondria-associated processes in animal models of MIRI.

### 4.1 Comparison with previous meta-analyses

At the level of therapeutic efficacy, acupuncture preconditioning significantly reduced myocardial infarct size, a finding consistent with previous animal studies and clinical meta-analyses (10–12), further confirming the protective effects of acupuncture preconditioning against reperfusion injury. Compared with prior meta-analyses, the study by Yang et al., although based on animal experiments, was restricted to electroacupuncture at a single acupoint (PC6) and focused primarily on electrocardiographic and myocardial enzyme outcomes. The investigations by Xiong et al. and Qin et al., both being clinical studies, were unable to provide mechanistic evidence at the tissue and molecular levels. None of these previous reviews systematically synthesized evidence from animal studies specifically from the perspective of mitochondrial function, a gap that the present study sought to address.

### 4.2 Acupoint selection and compatibility patterns

In terms of acupoint selection, PC6 as a monotherapy was the most frequently employed regimen. PC6 is the Luo-connecting point of the Pericardium Meridian, one of the eight confluent points that communicates with the Yin Wei Meridian, and is traditionally regarded as a key acupoint for treating cardiac and thoracic disorders. Previous reviews have systematically summarized the protective mechanisms of PC6 in MIRI, suggesting that it modulates cardiac function and confers myocardial protection through the neuro-humoral-immune network (54). Clinical randomized controlled trials have further demonstrated that electroacupuncture preconditioning at PC6 reduces serum cTnI levels, shortens ICU length of stay, and alleviates oxidative stress injury in patients undergoing cardiac valve replacement surgery (55, 56).

Regarding acupoint compatibility, the combination of PC6 with CV4 and ST36 was the most commonly reported regimen. Previous studies have shown that electroacupuncture at PC6 and ST36 reduces infarct size following MIRI, and ST36 is frequently used in conjunction with PC6 to enhance cardioprotective effects (57). However, the limited number of studies for each compatibility regimen precludes a definitive conclusion regarding the optimal acupoint combination, which warrants further investigation.

### 4.3 Potential mechanistic interpretation

Mitochondrial dysfunction represents a critical juncture in the transition from reversible to irreversible myocardial injury during MIRI (58, 59). The protective effects of acupuncture preconditioning appear to act on distinct phases of this pathological process.

During ischemia, mitochondrial oxidative phosphorylation is suppressed, resulting in diminished ATP synthesis. Upon reperfusion, mitochondrial Ca^2^⁺ overload triggers the irreversible opening of the mitochondrial permeability transition pore (mPTP) (60). Once the mPTP opens, the inner mitochondrial membrane permeability increases abruptly, leading to uncoupling of oxidative phosphorylation and a shift in mitochondrial function from ATP synthesis to ATP hydrolysis, which culminates in dissipation of the membrane potential and further ATP depletion (61). The present study observed a significant restoration of myocardial ATP content following acupuncture. One possible explanation is that acupuncture preserves mitochondrial membrane integrity by inhibiting mPTP opening, as proposed in previous experimental studies.

Another critical event during early reperfusion is the burst of ROS. Elevated ROS levels activate the mitochondrial apoptotic pathway through multiple mechanisms. With respect to DNA damage, ROS can directly oxidize mitochondrial DNA, manifested as increased 8-hydroxydeoxyguanosine levels, decreased mitochondrial DNA copy number, and reduced transcriptional activity, with OGG1 and MYH playing synergistic roles in the repair process (62, 63). Concurrently, ROS initiate a chain reaction of lipid peroxidation in the mitochondrial membrane, generating reactive aldehydes such as 4-hydroxynonenal, which can covalently modify subunits of the respiratory chain complexes and interfere with electron transport (64). Recent work by Mendoza et al. has further demonstrated that simultaneous intervention targeting both mPTP opening and lipid peroxidation yields additive cardioprotective effects, suggesting a synergistic pathogenic contribution of these two processes in ischemia-reperfusion injury (65). The concurrent reductions in ROS and MDA observed in the present study suggest that acupuncture may attenuate oxidative stress injury during early reperfusion, thereby participating in the regulation of the initiation signals of the apoptotic pathway.

In the context of ROS-mediated mitochondrial injury, Bax and Bcl-2 constitute the core regulatory nodes of the mitochondrial apoptotic pathway. Bcl-2, anchored in the outer mitochondrial membrane, blocks pore formation by directly binding to Bax and inhibiting its pro-apoptotic oligomerization. In contrast, Bax translocates from the cytosol to the mitochondrial membrane upon apoptotic stimulation, promoting mitochondrial outer membrane permeabilization (66). The present study observed upregulation of Bcl-2 protein and downregulation of Bax protein—a coordinated bidirectional shift that collectively suggests a reduced propensity of mitochondria to respond to apoptotic stimuli, through both suppression of pro-apoptotic signaling and enhancement of anti-apoptotic capacity. Beyond apoptosis regulation, acupuncture may also modulate excessive mitophagy mediated by the mTORC1-ULK1-FUNDC1 pathway, thereby maintaining the clearance of damaged mitochondria at an appropriate level conducive to cell survival (8).

From molecular events to cellular outcomes, the reduction in the apoptotic index suggests that acupuncture may interrupt the transmission from molecular activation to apoptotic execution. Ultimately, at the tissue level, the reduction in infarct size likely reflects the integrated effects of energy restoration, oxidative scavenging, and apoptosis suppression.

These mechanistic interpretations should not be regarded as exclusive. Other pathways, such as ferroptosis, necroptosis, endoplasmic reticulum stress, and m6A regulation, may also contribute to the observed outcomes. Therefore, the observed effects should not be attributed exclusively to mitochondrial protection.

### 4.4 Heterogeneity, evidence quality, and limitations

Heterogeneity was generally high across all outcome measures, which is a common phenomenon in meta-analyses of animal studies. Subgroup analyses did not adequately explain the observed heterogeneity. Although species and reperfusion duration significantly modified the effect for Bcl-2, within-subgroup heterogeneity remained high in most strata. For the remaining outcomes, no analyzed subgroup factor significantly modified the effect sizes, and heterogeneity persisted. These findings suggest that heterogeneity may arise from other sources not captured by the analyzed subgroup factors, such as variations in acupuncture parameters or other unanalyzed aspects of model preparation, including surgical procedures, anesthesia, and criteria for model success. However, while the statistical significance of the pooled results was robust, sensitivity analyses revealed that the extreme magnitudes of effect sizes for ROS, Bax, Bcl-2, and AI were heavily driven by a few individual studies. These studies either reported extremely small standard deviations—which mathematically overestimates the SMD—or used different measurement scales. Therefore, the true effect sizes of these apoptotic markers are likely more conservative than the pooled estimates. Exploratory sensitivity analyses by publication language and research team clustering further showed that infarct size, ATP, Bcl-2, and AI were relatively robust, whereas MDA, Bax, and ROS were sensitive to specific clusters, suggesting that the pooled estimates for these three outcomes should be interpreted with caution.

GRADE ratings indicated that the certainty of evidence was moderate to low, primarily due to risk of bias, as most studies were rated as unclear in sequence generation, allocation concealment, and blinding according to the SYRCLE assessment. Bax was further downgraded for imprecision, given its lowest total sample size (*N* = 86) and widest confidence interval among all outcomes. Publication bias was not consistently detected; for ROS, trim-and-fill suggested one potentially missing study, but this was not considered sufficient to downgrade. It should be noted that the moderate-to-low certainty primarily reflects methodological limitations of the primary studies rather than implausibility of the acupuncture effects themselves.

The following limitations should be acknowledged. First, all included studies were animal experiments, and caution is warranted when extrapolating the findings to clinical practice. Second, the primary analysis used untreated model controls; sham-controlled data were too sparse to distinguish acupoint-specific from non-specific effects. Third, the methodological quality of the primary studies was generally suboptimal. In addition, the PROSPERO registration was retrospective with respect to the search stage; however, no eligibility criteria, primary outcomes, or primary analysis plan were changed after registration, and the eligibility criteria were more restrictive than the registered protocol. Fourth, despite considerable variation in acupuncture parameters across studies, the limited number of studies precluded comprehensive subgroup analyses to examine interactions among these factors. Fifth, although publication bias was not consistently detected, the possibility of small-study effects cannot be entirely excluded, particularly for outcomes with fewer than ten studies where formal assessment was not performed. Sixth, although 95% PIs were calculated, all outcomes included only 7–13 studies (K < 15). According to Matrai et al. (67), the coverage of a single PI may deviate substantially from the nominal level in this setting; therefore, the 95% PIs should be regarded as exploratory supplementary information and not used for primary inference. Seventh, the extreme pooled effect sizes for ROS, Bax, Bcl-2, and AI may have been statistically overestimated. This is primarily due to the inclusion of primary studies reporting unusually small standard deviations or employing different measurement scales. Consequently, these specific results should be interpreted as exploratory rather than definitive. Eighth, there is a translational gap regarding outcome assessment. The parameters evaluated in this review are myocardial tissue readouts, which are not routinely accessible in clinical practice. Circulating biomarkers were not included in this review by design, and no blood-based biomarker data were pooled. Therefore, the present findings cannot directly guide the selection of circulating biomarkers. Future biomarker studies should be restricted to planned/elective settings in which preconditioning is feasible (e.g., elective cardiac surgery), rather than unanticipated STEMI. Finally, direct mitochondrial readouts, including mitochondrial membrane potential, mPTP opening, and respiratory activity, were not quantitatively synthesized. The mitochondrial hypothesis examined here therefore relies on indirect surrogate outcomes and cannot be regarded as established. Graphical data were also digitized once and verified by a second investigator; this protocol deviation may introduce measurement error.

Despite these limitations, the present findings suggest that acupuncture preconditioning holds promise as a feasible adjunctive strategy for myocardial protection in planned/elective cardiac procedures, given its non-invasive nature, low cost, and favorable safety profile.

## 5 Conclusion

Although the present study is based on preclinical animal experiments, its findings may inform future mechanistic studies in planned/elective settings. However, they cannot directly guide circulating biomarker selection because the pooled outcomes were tissue-based.

In summary, electroacupuncture preconditioning was associated with reduced myocardial injury in animal models of MIRI and with favorable changes in mitochondria-associated parameters. Only one manual acupuncture study was included; these findings therefore primarily reflect electroacupuncture and should not be generalized to acupuncture as a whole. However, the extreme effect sizes for ROS, Bax, Bcl-2, and AI may be statistically overestimated due to small SDs or methodological scale differences, rendering these findings hypothesis-generating. Given the moderate-to-low certainty of evidence and sparse sham-controlled data, higher-quality animal studies with sham or non-acupoint controls are needed, and clinical extrapolation should be made with caution.

## Data Availability

The original contributions presented in the study are included in the article/Supplementary Material, further inquiries can be directed to the corresponding author.
