# Supplementary material for: Electroacupuncture preconditioning and mitochondria-associated outcomes in animal models of myocardial ischemia-reperfusion injury: a systematic review and meta-analysis
Source: Front Cardiovasc Med. 2026 Oct 30;13:1982708. doi: 10.3389/fcvm.2026.1982708 (PMC13759788; doi:10.3389/fcvm.2026.1982708)
Supplement: Supplementary file 1 [file Supplementaryfile1.docx]

**Additional file 1: Search strategies**

**Pubmed**：(("Acupuncture Therapy"[Mesh]) OR (Acupuncture Treatment[Title/Abstract] OR Acupuncture Treatments[Title/Abstract] OR Treatment, Acupuncture[Title/Abstract] OR Therapy, Acupuncture[Title/Abstract] OR Pharmacoacupuncture Treatment[Title/Abstract] OR Treatment, Pharmacoacupuncture[Title/Abstract] OR Pharmacoacupuncture Therapy[Title/Abstract] OR Therapy, Pharmacoacupuncture[Title/Abstract] OR Acupotomy[Title/Abstract] OR Acupotomies[Title/Abstract])) AND (("Myocardial Reperfusion Injury"[Mesh]) OR (Injuries, Myocardial Reperfusion[Title/Abstract] OR Myocardial Reperfusion Injuries[Title/Abstract] OR Reperfusion Injuries, Myocardial[Title/Abstract] OR Reperfusion Injury, Myocardial[Title/Abstract] OR Injury, Myocardial Reperfusion[Title/Abstract] OR Myocardial Ischemic Reperfusion Injury[Title/Abstract]))

**Web of Science:**

1: Myocardial Reperfusion Injury (Topic)

2: AB=(Injuries, Myocardial Reperfusion or Myocardial Reperfusion Injuries or Reperfusion Injuries, Myocardial or Reperfusion Injury, Myocardial or Injury, Myocardial Reperfusion or Myocardial Ischemic Reperfusion Injury)

3: #2 OR #1

4: (TS=(Acupuncture Therapy)) OR AB=(Acupuncture Treatment or Acupuncture Treatments or Treatment, Acupuncture or Therapy, Acupuncture or Pharmacoacupuncture Treatment or Treatment, Pharmacoacupuncture or Pharmacoacupuncture Therapy or Therapy, Pharmacoacupuncture or Acupotomy or Acupotomies)

5: #3 AND #4

**Cochranelibrary：**

#1	MeSH descriptor: [Acupuncture Therapy] explode all trees

#2	MeSH descriptor: [Myocardial Reperfusion Injury] explode all trees

#3	(Acupuncture Treatment):ti,ab,kw OR (Acupuncture Treatments):ti,ab,kw OR (Treatment, Acupuncture):ti,ab,kw OR (Therapy, Acupuncture):ti,ab,kw OR (Pharmacoacupuncture Treatment):ti,ab,kw OR (Treatment, Pharmacoacupuncture):ti,ab,kw OR (Pharmacoacupuncture Therapy):ti,ab,kw OR (Therapy, Pharmacoacupuncture):ti,ab,kw OR (Acupotomy):ti,ab,kw OR (Acupotomies):ti,ab,kw

#4	(Injuries, Myocardial Reperfusion):ti,ab,kw OR (Myocardial Reperfusion Injuries):ti,ab,kw OR (Reperfusion Injuries, Myocardial):ti,ab,kw OR (Reperfusion Injury, Myocardial):ti,ab,kw OR (Injury, Myocardial Reperfusion):ti,ab,kw OR (Myocardial Ischemic Reperfusion Injury):ti,ab,kw

#5	#1 or #3

#6	#2 or #4

#7	#5 and #6

**Embase：**#7：#13 AND #10

#6：#11 OR #12

#5：'acupuncture therapy':ab,ti OR 'shonishin':ab,ti OR 'acupuncture':ab,ti

#4：'acupuncture'/exp

#3：#9 OR #8

#2：((((((((('cardiac ir injury':ab,ti AND reperfusion:ab,ti OR 'cardiac iri':ab,ti OR 'cardiac ischaemia reperfusion injury':ab,ti OR 'cardiac ischaemia/reperfusion':ab,ti) AND ir:ab,ti AND injury:ab,ti OR 'cardiac ischaemic reperfusion injury':ab,ti OR 'cardiac ischemia reperfusion injury':ab,ti OR 'cardiac ischemia/reperfusion':ab,ti) AND ir:ab,ti AND injury:ab,ti OR 'cardiac ischemic reperfusion injury':ab,ti OR 'cardiac reperfusion injury':ab,ti OR 'cardiomyocyte ir injury':ab,ti) AND reperfusion:ab,ti OR 'cardiomyocyte iri':ab,ti OR 'cardiomyocyte ischaemia-reperfusion injury':ab,ti OR 'cardiomyocyte ischemia-reperfusion injury':ab,ti OR 'heart ir injury':ab,ti) AND reperfusion:ab,ti OR 'heart iri':ab,ti OR 'heart ischaemia reperfusion injury':ab,ti OR 'heart ischaemia-reperfusion':ab,ti) AND ir:ab,ti AND injury:ab,ti OR 'heart ischaemic reperfusion injury':ab,ti OR 'heart ischemia reperfusion injury':ab,ti OR 'heart ischemia-reperfusion':ab,ti) AND ir:ab,ti AND injury:ab,ti OR 'heart ischemic reperfusion injury':ab,ti OR 'heart muscle ischemia reperfusion injury':ab,ti OR 'heart muscle ischemic reperfusion injury':ab,ti OR 'heart reperfusion injury':ab,ti OR 'myo-cardial ischemia-reperfusion injury':ab,ti OR 'myo-cardial ischemic-reperfusion injury':ab,ti OR 'myocardial ir injury':ab,ti) AND reperfusion:ab,ti OR 'myocardial iri':ab,ti OR 'myocardial ischaemia reperfusion injury':ab,ti OR 'myocardial ischaemia-reperfusion':ab,ti) AND ir:ab,ti AND injury:ab,ti OR 'myocardial ischaemic reperfusion injury':ab,ti OR 'myocardial ischemia-reperfusion':ab,ti) AND ir:ab,ti AND injury:ab,ti OR 'myocardial ischemic reperfusion injury':ab,ti OR 'myocardial reperfusion injury':ab,ti OR 'myocardium ir injury':ab,ti OR 'myocardium iri':ab,ti OR 'myocardium ischaemia reperfusion injury':ab,ti OR 'myocardium ischaemic reperfusion injury':ab,ti OR 'myocardium ischemia reperfusion injury':ab,ti OR 'myocardium ischemic reperfusion injury':ab,ti OR 'myocardium reperfusion injury':ab,ti OR 'reperfusion cardiac injury':ab,ti OR 'reperfusion heart injury':ab,ti OR 'reperfusion myocardial injury':ab,ti OR 'reperfusion-induced cardiac injury':ab,ti OR 'reperfusion-induced cardiomyocyte injury':ab,ti OR 'reperfusion-induced heart injury':ab,ti OR 'reperfusion-induced myocardial injury':ab,ti OR 'myocardial ischemia reperfusion injury':ab,ti

#1：'myocardial ischemia reperfusion injury'/exp

**China National Knowledge Infrastructure：**（主题：针灸疗法 + 针刺 + 针灸 + 电针）AND（主题：心肌再灌注损伤 + 缺血心肌再灌注损伤 + 心肌缺血再灌注损伤）

English translation: ((Subject: acupuncture therapy + acupuncture + acupuncture and moxibustion + electroacupuncture) AND (Subject: myocardial reperfusion injury + ischemic myocardial reperfusion injury + myocardial ischemia-reperfusion injury))

**Chinese Biomedical Literature Database**：(("针刺"[常用字段:智能] OR "针灸"[常用字段:智能] OR "电针"[常用字段:智能]) OR ("针灸疗法"[不加权:扩展])) AND (("缺血心肌再灌注损伤"[常用字段:智能] OR "心肌缺血再灌注损伤"[常用字段:智能]) OR ("心肌再灌注损伤"[不加权:扩展]))

English translation: (("Acupuncture"[Common Field: Smart] OR "Acupuncture and Moxibustion"[Common Field: Smart] OR "Electroacupuncture"[Common Field: Smart]) OR ("Acupuncture Therapy"[Unweighted: Expanded])) AND (("Ischemic Myocardial Reperfusion Injury"[Common Field: Smart] OR "Myocardial Ischemia-Reperfusion Injury"[Common Field: Smart]) OR ("Myocardial Reperfusion Injury"[Unweighted: Expanded]))

**VIP Chinese Science and Technology Periodical Database**：题目或关键词 = 针灸疗法 + 针刺 + 针灸 + 电针 AND 题目或关键词 = 心肌再灌注损伤 + 缺血心肌再灌注损伤 + 心肌缺血再灌注损伤）

English translation: ((Title or Keyword = acupuncture therapy OR acupuncture OR acupuncture and moxibustion OR electroacupuncture) AND (Title or Keyword = myocardial reperfusion injury OR ischemic myocardial reperfusion injury OR myocardial ischemia-reperfusion injury))

**Wanfang：**主题:(针灸疗法 or 针刺 or 针灸 or 电针) and 主题:(心肌再灌注损伤 or 缺血心肌再灌注损伤 or 心肌缺血再灌注损伤)

English translation: Subject:(acupuncture therapy OR acupuncture OR acupuncture and moxibustion OR electroacupuncture) AND Subject:(myocardial reperfusion injury OR ischemic myocardial reperfusion injury OR myocardial ischemia-reperfusion injury)
