# Supplementary material for: Electroacupuncture preconditioning and mitochondria-associated outcomes in animal models of myocardial ischemia-reperfusion injury: a systematic review and meta-analysis
Source: Front Cardiovasc Med. 2026 Oct 30;13:1982708. doi: 10.3389/fcvm.2026.1982708 (PMC13759788; doi:10.3389/fcvm.2026.1982708)
Supplement: Supplementary file 3 [file Table5.docx]

**Question:** Acupuncture preconditioning compared to model control for myocardial ischemia-reperfusion injury in animal models

**Setting:** Preclinical animal study

| **Certainty assessment** | | | | | | | **№ of patients** | | **Effect** | | **Certainty** | **Importance** |
| --- | --- | --- | --- | --- | --- | --- | --- | --- | --- | --- | --- | --- |
| **№ of studies** | **Study design** | **Risk of bias** | **Inconsistency** | **Indirectness** | **Imprecision** | **Other considerations** | **acupuncture preconditioning** | **model control** | **Relative (95% CI)** | **Absolute (95% CI)** |  |  |
| **MDA** | | | | | | | | | | | | |
| 7 | randomised trials | serious^a^ | not serious | not serious | not serious | none^b^ | 52 | 52 | - | SMD **3.76 SD lower** (6.22 lower to 1.29 lower) | ⨁⨁⨁◯ Moderate^a,b^ | CRITICAL |
| **ATP** | | | | | | | | | | | | |
| 8 | randomised trials | serious^a^ | not serious | not serious | not serious | none^b^ | 74 | 74 | - | SMD **3.21 SD higher** (2.12 higher to 4.29 higher) | ⨁⨁⨁◯ Moderate^a,b^ | CRITICAL |
| **ROS** | | | | | | | | | | | | |
| 10 | randomised trials | serious^a^ | not serious | not serious | not serious | none^c^ | 88 | 88 | - | SMD **4.02 SD lower** (7.17 lower to 0.87 lower) | ⨁⨁⨁◯ Moderate^a,c^ | CRITICAL |
| **Bax** | | | | | | | | | | | | |
| 7 | randomised trials | serious^a^ | not serious | not serious | serious^d^ | none^b^ | 43 | 43 | - | SMD **6.52 SD lower** (11.43 lower to 1.62 lower) | ⨁⨁◯◯ Low^a,b,d^ | CRITICAL |
| **Bcl-2** | | | | | | | | | | | | |
| 10 | randomised trials | serious^a^ | not serious | not serious | not serious | none^e^ | 65 | 65 | - | SMD **8.14 SD higher** (3.82 higher to 12.45 higher) | ⨁⨁⨁◯ Moderate^a,e^ | CRITICAL |
| **AI** | | | | | | | | | | | | |
| 13 | randomised trials | serious^a^ | not serious | not serious | not serious | none^e^ | 102 | 98 | - | SMD **6.36 SD lower** (10.35 lower to 2.38 lower) | ⨁⨁⨁◯ Moderate^a,e^ | CRITICAL |
| **infarct size** | | | | | | | | | | | | |
| 13 | randomised trials | serious^a^ | not serious | not serious | not serious | none^e^ | 101 | 98 | - | SMD **2.65 SD lower** (3.69 lower to 1.61 lower) | ⨁⨁⨁◯ Moderate^a,e^ | IMPORTANT |

**CI:** confidence interval; **SMD:** standardised mean difference

#### Explanations

a. Risk of bias was judged as serious. Most of the included studies were rated as ‘unclear’ risk in key domains (e.g., allocation concealment, blinding) using the SYRCLE tool, indicating potential methodological limitations that may overestimate the intervention effect.

b. Publication bias was not formally assessed because fewer than 10 studies were available for this outcome; it was therefore rated as undetected, not assumed to be absent.

c. Egger's test did not suggest significant publication bias. Trim-and-fill analysis estimated one potentially missing study and the adjusted estimate became non-significant; however, given the high heterogeneity and non-significant Egger's test, this was not considered sufficient evidence to downgrade for publication bias.

d. Total sample size (N=86) below OIS; 95% CI [-11.43, -1.62] is extremely wide, indicating substantial uncertainty in the effect magnitude.

e. Egger's test did not suggest significant publication bias (see Table 1).
